# Radiostereometric analysis for evaluating inducible fracture micromotion: a scoping review

**DOI:** 10.2340/17453674.2025.44897

**Published:** 2025-10-27

**Authors:** Michaela Manalili HANSEN, Mohammad Laith BALLO, Stephan Maximillian RÖHRL

**Affiliations:** 1 Department of Orthopaedic Surgery, Odense University Hospital, Odense; 2 Institute of Clinical Medicine, University of Southern Denmark, Odense, Denmark; 3 Division of Orthopaedic Surgery, Oslo University Hospital, Oslo; 4 Institute of Clinical Medicine, Faculty of Medicine, University of Oslo, Oslo, Norway

## Abstract

**Background and purpose:**

Reliable assessment of fracture healing remains a clinical challenge as radiographs and clinical examination provide only indirect information. Inducible fracture micromotion, defined as fragment displacement under load, may offer a more direct surrogate for healing. Radiostereometric analysis (RSA) can measure micromotion with high precision, but its clinical use for fracture assessment remains limited and heterogeneous. This scoping review aimed to map the existing literature on RSA for inducible fracture micromotion and summarize methodological approaches to guide future research.

**Methods:**

We systematically searched Medline, Embase, and Scopus. Clinical studies applying RSA to assess inducible fracture micromotion were eligible. 2 reviewers independently screened and extracted data on study design, patient population, fracture location, loading protocols, thresholds for motion, and outcomes.

**Results:**

7 clinical studies were included, comprising feasibility studies, prospective cohorts, and 1 imaging study. Sample sizes ranged from 6 to 16 patients, with fractures of the distal radius, femur, proximal tibia, and pelvis. All studies required intraoperative implantation of tantalum markers. Most applied differentially loaded RSA, typically comparing unloaded and loaded conditions using weightbearing platforms, force plates, or voluntary grip dynamometry. Despite varied protocols and small, single-center designs, RSA consistently detected small-scale inducible motion and, in some studies, distinguished union from non-union.

**Conclusion:**

This scoping review identified 7 clinical studies using RSA to assess inducible fracture micromotion, with heterogeneous methods across fracture types. These findings may guide the development of standardized approaches and support future research on RSA in fracture healing.

Assessment of fracture healing remains a fundamental aspect of orthopedic trauma care. In clinical practice, fracture union is typically evaluated by sequential radiographs and clinical examination [1]. Radiographic signs such as callus formation and cortical bridging are indirect healing markers, while the absence of pain during weightbearing is often considered a clinical sign of union [2]. However, both modalities have limitations. Radiographs are semiquantitative, subject to interpretation, and frequently delayed in detecting non-union [3]. Likewise, clinical evaluation is highly subjective, and no consensus exists on standardized diagnostic criteria for healing or failure [2]. Mechanical stability is essential for fracture healing, but most current methods to assess fracture healing do not reflect the mechanical stability of the fracture site [4].

Inducible motion—defined as micromotion under load—has been used to assess implant fixation in joint arthroplasty with RSA [5,6]. In contrast, its use in fracture care remains limited. RSA has been introduced as an accurate method for assessing micromotion between fracture fragments [7].

Despite promising results, no standardized clinical framework exists for using inducible fracture micromotion in research or to guide fracture management. Published studies vary widely in fracture type, fixation method, loading protocol, and outcome measures, making comparison and interpretation challenging. Because the literature is sparse and heterogeneous, a scoping review is particularly suited to mapping existing evidence, clarifying methodological diversity, and identifying gaps to guide future studies [8].

We therefore conducted a scoping review of clinical studies using RSA to assess inducible fracture micromotion, with the aim of providing an overview of study designs, fracture types, loading protocols, and outcome measures. Our objective was to map current methodologies, describe the settings in which inducible motion has been applied, and highlight gaps that may inform the development of standardized approaches and future clinical research.

## Methods

### Protocol and registration

A scoping review approach was chosen to capture the breadth of available evidence, accommodate heterogeneous study designs, and identify methodological gaps rather than assess study quality. The protocol was not preregistered. Reporting follows the PRISMA-ScR extension of the PRISMA guidelines [9].

### Eligibility criteria

We included studies that employed any form of RSA to evaluate inducible fracture migration. Only clinical studies were eligible. Eligible sources included peer-reviewed journal articles and conference abstracts presenting original data.

We excluded studies that investigated implant migration without concurrent assessment of fracture stability, editorials, narrative reviews, expert opinions, and studies using non-RSA modalities to assess fracture motion.

### Information sources and search strategy

In May 2025, we conducted a comprehensive literature search in Medline, Embase, and Scopus. Medline and Embase were selected because they cover clinical, orthopedic, and device-related literature relevant to fracture healing and RSA. Scopus was included to capture additional studies from engineering and interdisciplinary journals that were not indexed in Medline or Embase.

Search strategies were tailored to each database and are provided in Supplementary data. We validated the search string by confirming that it retrieved at least 3 key studies relevant to the review topic [7-9]. Additionally, reference lists of all included studies were screened manually to identify relevant studies not captured by the database search.

### Selection of sources of evidence

Screening was conducted using Covidence software (Veritas Health Innovation, Melbourne, Australia).

2 reviewers (MH, MB) independently screened titles and abstracts to identify potentially eligible studies, applying a liberal inclusion approach during the initial screening phase. Full-text articles were then assessed for final inclusion. Discrepancies between reviewers were resolved through discussion; if consensus could not be reached, a third reviewer was consulted.

### Data charting process

Data were charted using a standardized form developed by the research team. 1 reviewer (MH) performed the initial data extraction, which was then independently verified by a second reviewer (MB) to ensure accuracy and completeness. The charting form was pilot-tested on a subset of studies to ensure consistency.

### Data items

The following data items were extracted from each included study: study characteristics (first author, publication year, country, area of body, study design, number of patients), term for method, threshold for migration, inducement method, and key findings.

### Critical appraisal and synthesis of results

A formal quality appraisal of included studies was not conducted, as this falls outside the scope of a scoping review. Instead, data was charted, and results were organized in tables where applicable, categorizing studies by fracture type, RSA methodology, outcome measures, and conclusions.

### Ethics, registration, data sharing plan, funding, use of AI tools, and disclosures

No ethical approval was needed. Data is available upon reasonable request through the corresponding author. This review is supported by the OUH Internalization Fund. The funder has no role in the design, analysis, data interpretation, or decision to submit results. During the preparation of this work the authors used Grammarly (Grammarly, Inc. 2025) and Scite.ai as tools in order to improve readability and check grammar/spelling as well as to check references. After using this tool/service, the authors reviewed and edited the content as needed and take full responsibility for the content of the publication.

No competing interests are reported. Complete disclosure of interest forms according to ICMJE are available on the article page, doi: 10.2340/17453674.2025.44897

## Results

### Selection of sources of evidence

The search yielded 364 records, of which 151 were identified as duplicates using Covidence software. The remaining 213 records were screened by title and abstract. An additional 4 duplicates were identified manually. 31 studies underwent full-text review. Following full-text screening, 7 studies met the inclusion criteria [10-16]. In addition, 1 study protocol and 2 pre-clinical trials were identified [17,18] (Figure).

**Figure:**
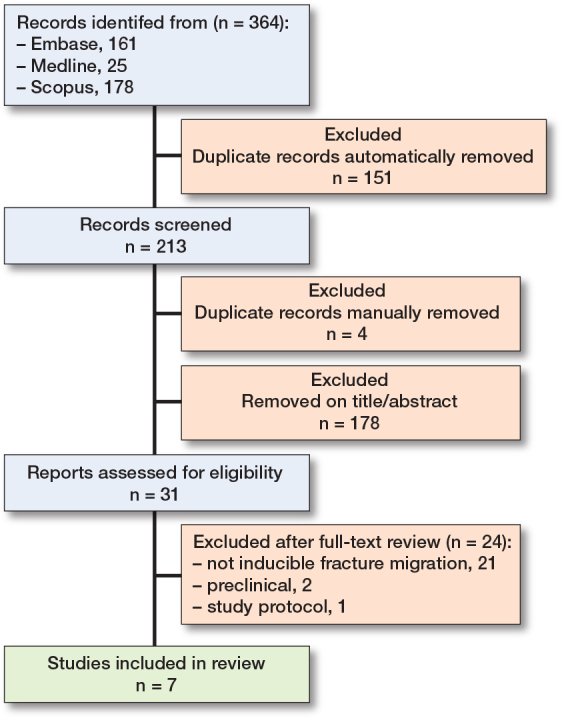
PRISMA flowchart.

### Characteristics of sources of evidence

7 clinical studies were included. The studies originated from Australia (n = 3), Finland (n = 2), Scotland (n = 1), and the USA (n = 1). Study designs included feasibility studies (n = 3), prospective cohort studies (n = 3), and 1 prospective imaging study. Anatomical locations varied and included distal radius (n = 2), distal femur (n = 2), proximal femur (n = 1), proximal tibia (n = 1), and pelvic ring (n = 1). Sample sizes ranged from 6 to 16 patients. All studies applied RSA, with 5 employing differentially loaded RSA, 1 using dynamic inducible micromotion, and 1 using RSA with voluntary grip loading to assess inducible motion under load. In all studies, tantalum markers were implanted intraoperatively, and RSA imaging was performed in both unloaded and loaded conditions to quantify motion during healing.

### Results of individual sources of evidence

RSA was feasible across all anatomical sites and provided information not visible on standard radiographs (Table 1). Chehade et al. [10] was the only study to apply incremental weightbearing; all others assessed micromotion under a single applied load. Thresholds ranged from 0.00–3.95 mm for translation and 0.00–3.00 degrees for rotation in absolute values. Reported thresholds and definitions of motion differed, but each study demonstrated the capacity of RSA to quantify inducible fracture micromotion during healing.

**Table 1:** Study characteristics and feasibility

First author (year)	Country	Fracture type	Study design	n	Term for method	Inducement method	Feasibility
Chehade (2009) [10]	Australia	Distal femur	Prospective feasibility study	6	DLRSA	Standing weightbearing (20–60 kg) using digital scales	DLRSA was feasible and introduced as a novel method to assess healing
Downing (2008) [11]	Scotland	Distal radius	Prospective imaging feasibility study	9	Dynamic inducible micromotion	Maximal voluntary grip using a dynamometer	RSA was feasible for tracking healing in volar plate constructs
Finnilä (2018) [12]	Finland	Proximal femur	Prospective exploratory cohort study	16	DLRSA	Supine axial loading with foot pressing on force plate	DLRSA was feasible and may help identify unstable fixations
Galea (2020) [13]	USA	Distal femur	Prospective cohort study	16	DLRSA	Supine axial load up to 178 N via force plate	DLRSA was feasible and provided objective data on fracture stability
Ladurner (2020) [14]	Australia	Pelvic ring	Prospective	6	DLRSA	Standing weightbearing	DLRSA was feasible for assessing postoperative pelvic ring stability
Madanat (2012) [15]	Finland	Distal radius	Prospective cohort study	15	RSA with voluntary grip loading	Maximal voluntary grip (rubber ball)	RSA method was feasible but technically challenging
Solomon (2011) [16]	Australia	Proximal tibia	Case series	7	DLRSA	Standing weightbearing on calibrated scale	DLRSA was feasible for evaluating tibial plateau fragment stability

DLRSA: differentially loaded radiostereometric analysis; RSA: radiostereometric analysis.

### Synthesis of results

Table 2 presents quantitative findings. Across studies, RSA consistently detected small-scale inducible micromotion, which typically decreased over time, while persistence was associated with delayed or non-union. Where clinical or patient-reported outcomes were reported, they generally aligned with RSA findings [12-15].

**Table 2:** Study results

First author (year)	Micromotion		Threshold	Condition number (range)	Micromotion over time	Patient-reported outcomes
	range, mm	range, °				
Chehade (2009) [10]	0.00–3.95	0.0–6.6	NR	84 (38–142)	Micromotion decreased over time except in 1 non-union	No PROMs collected
Downing (2008) [11]	< 0.3 **^a^**	< 2.5 **^a^**	NR	124 (89–408) **^d^** 118 (72–208) **^e^**	Micromotion peaked at 2 weeks and resolved by 26 weeks	No PROMs collected
Finnilä (2018) [12]	0.27–0.39	0.36–1.17	≥ 0.3 mm or ≥ 1.2°	113 (61–244) **^d^** 109 (57–192) **^e^**	Micromotion resolved by 12 weeks in unions but persisted in non-unions	Pain VAS during loading was recorded but not correlated to RSA findings
Galea (2020) [13]	0.03–3.4 **^b^**	0.03–1.03 **^b^**	≥ 0.9 mm	26 ± 10	Micromotion decreased over time but some persisted at 12 months	Pain and functional improvements paralleled the increasing fracture stiffness as measured by RSA
Ladurner (2020) [14]	< 5.7 **^c^**	< 3 **^c^**	NR	< 150	Progressive decrease over time, approaching zero at 12 months	The patients with the most inducible fracture micromotion had the worst Iowa Pelvic Scores
Madanat (2012) [15]	0.01–0.19 **^c^**	0.01–1.29 **^c^**	≥ 0.10 mm or ≥ 1.01°	< 1,000	Detectable up to 18 weeks despite radiographic union	Although inducible micromotion persisted up to 18 weeks, clinical recovery improved steadily until 52 weeks
Solomon (2011) [16]	0.01–0.73 **^c^**	0.00–1.52 **^c^**	NR	NR	Translations/rotations decreased over time, and stabilized by 1 year	No PROMs collected

NR: not reported; PROMs: patient-reported outcome measures. VAS: visual analog scale. RSA: radiostereometric analysis.

a Max. median at 2 weeks

b Difference in mean

c Mean

d Object

e Reference

RSA demonstrated potential not only for assessing interfragmentary stability but also for predicting non-union. Several studies highlighted the feasibility of implementing RSA in a clinical setting despite technical barriers such as marker visibility or positioning. Fracture types varied, but the method was applied successfully to upper and lower extremity fractures and the pelvis.

## Discussion

This scoping review aimed to map the clinical literature on the use of RSA to measure inducible fracture micromotion. We identified 7 clinical studies that applied RSA to measure inducible fracture micromotion. Across fracture sites, RSA consistently detected small-scale motion, most often decreasing during healing, while persistence was linked to delayed or non-union. Importantly, some studies reported micromotion despite radiographic signs of healing, suggesting RSA may provide complementary biomechanical information. Where clinical outcomes were included, they generally aligned with RSA findings: higher micromotion was associated with worse function or pain, although reporting of patient outcomes was inconsistent and often limited. While most studies suggested that differentially loaded RSA can quantify in vivo fracture motion during loading, the evidence remains preliminary. The findings from the included studies tentatively support the use of RSA as a research tool for monitoring healing progression and could potentially help distinguish between union and non-union in a clinical setting.

However, the included studies were generally small, exploratory, and heterogeneous, making it difficult to make any firm conclusions.

An emerging modality, CT-based RSA (CT RSA), has been proposed as a promising alternative or complement to conventional marker-based RSA [19,20]. CT RSA offers high spatial resolution and does not require surgically implanted markers, which may simplify procedures and broaden anatomical applicability [21]. Although the use of CT RSA for measuring inducible fracture micromotion has not yet been demonstrated clinically, studies have confirmed its capacity to detect joint motion under load. For example, CT RSA has been used to assess midfoot kinematics with high precision compared with traditional RSA [22] and to evaluate motion preservation after Lisfranc injury treated with bridge plating [23]. Furthermore, a recent accuracy study comparing model-based RSA with an AI-driven CT RSA approach demonstrated promising results, supporting the method’s potential for precise, non-invasive motion tracking [20]. Key limitations include technical challenges with applying differential loading during CT acquisition, and further studies are needed to evaluate accuracy, reproducibility, radiation exposure, and clinical utility.

Dynamic RSA has been used to assess inducible micromotion in joint arthroplasty, reflecting fixation quality under physiological load [6]. Similarly, automated RSA techniques using CT-based models (AutoRSA) have shown high accuracy for implant migration and could be used to assess inducible migration without tantalum markers [24]. However, these methods have not yet been applied to inducible fracture micromotion and, despite their technical promise, clinical use in fracture settings remains unexplored.

Conventional RSA provides precise in vivo measurement of micro-motion and sensitivity to subtle biomechanical changes, making it well suited for research on fracture healing biomechanics. Its reliance on specialized equipment, trained personnel, and invasive marker implantation, however, restricts broader feasibility. At present, inducible fracture motion measurement with RSA remains primarily a research tool.

### Limitations

Limitations include the small number of eligible studies, and their small, exploratory, single-center design with limited follow-up cohorts. Most were feasibility or observational studies, and only 2 reported longitudinal clinical outcomes beyond imaging. Technical challenges related to marker placement and image clarity were commonly reported, particularly in anatomically complex regions like the distal radius and pelvis. Furthermore, heterogeneity in RSA protocols and loading conditions limited direct comparison between studies, which should be avoided in the future by following guidelines [7]. In addition, most studies provided limited follow-up and few clinical outcomes, restricting insight into the prognostic value of inducible micromotion. Pre-clinical studies and 1 protocol identified during screening were excluded from synthesis but noted for context.

### Conclusions

This scoping review mapped the available clinical literature on RSA for assessing inducible fracture micromotion. 7 small studies were identified, applying a range of loading protocols and methodological approaches across different fracture types. The review summarizes these approaches and illustrates the heterogeneity of current practice.

*In perspective,* these findings may serve as a basis for future work aiming to develop standardized methods and to explore the potential role of RSA in fracture research. Future studies should include larger, standardized designs with clinical endpoints to determine whether RSA-derived micromotion can predict healing and guide management. CT RSA may reduce invasiveness and expand applicability, though accessibility, radiation exposure, and validation under load-bearing conditions remain challenges.

### Supplementary data

Supplementary data with the search string is available on the article page, doi: 10.2340/17453674.2025.44897

## Supplementary Material



## Notes

MH: conceptualization, methodology, investigation, formal analysis, and writing—original draft. MB: investigation, formal analysis, and writing—review and editing. SR: Conceptualization, methodology, and writing—review and editing. All authors have read and approved the final version of the manuscript.

Handling co-editor: Bart Pijls

*Acta* thanks Hannu T Aro and other anonymous reviewers for help with peer review of this manuscript.
