# Supplementary material for: Radiostereometric analysis for evaluating inducible fracture micromotion: a scoping review
Source: Acta Orthop. 2025 Oct 27;96:822–7. doi: 10.2340/17453674.2025.44897 (PMC12559959; doi:10.2340/17453674.2025.44897)
Supplement: Supplementary file 1 [file ActaO-96-44897-s1.pdf]

Supplementary data

Radiostereometric Analysis for Evaluating Inducible Fracture Migration: A Scoping Review Protocol

Michaela Manalili Hansen [1,2], Mohammad Laith Ballo [3,4], and Stephan Maximillian Röhrl [3,4]

1 Department of Orthopaedic Surgery, Odense University Hospital, Odense, Denmark

2 Institute of Clinical Medicine, University of Southern Denmark, Odense, Denmark

3 Division of Orthopaedic Surgery, Oslo University Hospital, Oslo, Norway

4 Institute of Clinical Medicine, Faculty of Medicine, University of Oslo, Oslo, Norway

1. Background and rationale

Assessment of fracture healing remains a fundamental aspect of orthopedic trauma care. In clinical practice, fracture union is typically evaluated by sequential radiographs and clinical examination [1]. Radiographic signs such as callus formation and cortical bridging are indirect healing markers, while the absence of pain during weight-bearing is often considered a clinical sign of union [2]. However, both modalities have limitations: Radiographs are semiquantitative, subject to interpretation, and frequently delayed in detecting non-union [3]. Likewise, clinical evaluation is highly subjective, and no consensus exists on standardized diagnostic criteria for healing or failure [2]. Mechanical stability is essential for fracture healing, but most current methods to assess fracture healing do not reflect the biomechanical status of the healing site [4]. Inducible motion - defined as micromotion under load – has been used extensively to assess implant fixation in joint arthroplasty with RSA [5]. In contrast, its use in fracture care remains limited. RSA has been introduced as an accurate method for assessing micromotion between fracture fragments [6].

Despite promising results, no standardized clinical framework exists for using inducible fracture micromotion in research or to guide fracture management. Published studies vary widely in fracture type, fixation method, loading protocol, and outcome measures, making comparison and interpretation challenging**.** Because the literature is sparse and heterogeneous, a scoping review is particularly suited to mapping existing evidence, clarifying methodological diversity, and identifying gaps to guide future studies [7].

This protocol describes the methodology for a scoping review of clinical studies applying RSA to assess inducible fracture micromotion. The aim is to provide an overview of study designs, patient populations, loading protocols, and outcome measures, and to identify methodological gaps that may inform the development of standardized approaches and future clinical research.

2. Objectives

The primary objective of this scoping review is to identify and characterize studies that have utilized RSA to evaluate inducible fracture migration and micromotion published after 2005. Specific aims include:

1. Identifying the types of fractures evaluated for inducible fracture migration using RSA.
2. Characterizing the methodological approaches used for RSA data collection and analysis of inducible fracture migration.
3. Summarizing key findings regarding use of RSA to assess inducible fracture migration.
4. Identifying knowledge gaps and future research directions.

3. Methods

3.1 Eligibility Criteria

Inclusion Criteria:

- Studies utilizing any RSA modality to evaluate inducible fracture migration.
- Clinical studies
- Studies published in peer-reviewed journals and conference abstracts.

Exclusion Criteria:

- Studies focusing solely on implant migration without fracture assessment.
- Editorials or expert opinions without original data.
- Studies using non-RSA methods to assess fracture migration.
- Full text not available in English, Danish, Norwegian, or Swedish.
- Studies published before 2005.

3.2 Search Strategy

A systematic search will be conducted in the following databases:

- Medline
- Embase
- Scopus

Search terms for each database are listed in appendix 1.

The search string will not be limited by language restrictions, but will limit publication data to after 2005.

Reference lists of included studies will also be screened for additional relevant literature.

3.3 Study Selection

Two independent reviewers will screen titles and abstracts. Full-text reviews will be conducted for studies meeting inclusion criteria. Disagreements will be resolved through discussion or consultation with a third reviewer.

3.4 Data Extraction

A standardized data extraction form will be used to collect the following information:

- Study characteristics (authors, year, country, study design)
- Patient population or experimental model
- Type of fracture assessed
- RSA methodology (setup, follow-up intervals, analysis techniques)
- Reported outcomes

3.5 Data Synthesis

A narrative synthesis will be conducted to summarize study findings. Where applicable, a tabular summary will categorize study methodologies, fracture types, and RSA assessment techniques. Trends and gaps in the literature will be highlighted.

5. Ethical Considerations

As this study involves a review of existing literature, ethical approval is not required.

6. Dissemination Plan

Findings will be disseminated through publication in a peer-reviewed journal and presentation at relevant orthopaedic and/or radiology conferences.

7. Potential Limitations

- Variability in RSA methodologies across studies.
- Limited number of studies focusing specifically on inducible fracture migration/micromotion.
- Potential language bias in study selection.

8. Conclusion

This scoping review will provide a comprehensive overview of the application of RSA in assessing inducible fracture migration, identifying current evidence and research gaps to guide future investigations.

1. Bizzoca, D., Vicenti, G., Caiaffa, V., Abate, A., De Carolis, O., Carrozzo, M., Solarino, G., & Moretti, B. (2023). Assessment of fracture healing in orthopaedic trauma. *Injury*, *54 Suppl 1*, S46-s52. https://doi.org/10.1016/j.injury.2020.11.014
2. Corrales, L. A., Morshed, S., Bhandari, M., & Miclau, T., 3rd. (2008). Variability in the assessment of fracture-healing in orthopaedic trauma studies. *J Bone Joint Surg Am*, *90*(9), 1862-1868. https://doi.org/10.2106/jbjs.G.01580
3. Schwarzenberg, P., Darwiche, S., Yoon, R. S., & Dailey, H. L. (2020). Imaging Modalities to Assess Fracture Healing. *Curr Osteoporos Rep*, *18*(3), 169-179. https://doi.org/10.1007/s11914-020-00584-5
4. McClelland, D., Thomas, P. B., Bancroft, G., & Moorcraft, C. I. (2007). Fracture healing assessment comparing stiffness measurements using radiographs. *Clin Orthop Relat Res*, *457*, 214-219. https://doi.org/10.1097/BLO.0b013e31802f80a8
5. Cho, C. H., Pijls, B. G., Abrahams, J. M., Roerink, A., Katembwe, R., Baker, A., Solomon, L. B., & Callary, S. A. (2023). Migration patterns of acetabular cups: a systematic review and meta-analysis of RSA studies. *Acta Orthop*, *94*, 626-634. https://doi.org/10.2340/17453674.2023.24580
6. Kaptein, B. L., Pijls, B., Koster, L., Kärrholm, J., Hull, M., Niesen, A., Heesterbeek, P., Callary, S., Teeter, M., Gascoyne, T., Röhrl, S. M., Flivik, G., Bragonzoni, L., Laende, E., Sandberg, O., Solomon, L. B., Nelissen, R., & Stilling, M. (2024). Guideline for RSA and CT-RSA implant migration measurements: an update of standardizations and recommendations. *Acta Orthop*, *95*, 256-267. https://doi.org/10.2340/17453674.2024.40709
7. Munn, Z., Peters, M. D. J., Stern, C., Tufanaru, C., McArthur, A., & Aromataris, E. (2018). Systematic review or scoping review? Guidance for authors when choosing between a systematic or scoping review approach. *BMC Med Res Methodol*, *18*(1), 143. https://doi.org/10.1186/s12874-018-0611-x

Search string

Scopus

14/5/2025

(TITLE-ABS-KEY ("radiostereometric analysis" OR "radiostereometry")) AND (TITLE-ABS-KEY ("fracture*")) AND PUBYEAR > 2004 AND PUBYEAR < 2026

= 157 records

EMBASE

13/5/2025

1: exp radiostereometric analysis/ or radiostereometry.mp. [mp=title, abstract, heading word, drug trade name, original title, device manufacturer, drug manufacturer, device trade name, keyword heading word, floating subheading word, candidate term word] = 941

2: exp acetabulum fracture/ or exp ankle fracture/ or exp arm fracture/ or exp avulsion fracture/ or exp axis fracture/ or exp bimalleolar fracture/ or exp blowout fracture/ or exp burst fracture/ or exp calcaneus fracture/ or exp catheter fracture/ or exp cervical spine fracture/ or exp clavicle fracture/ or exp Colles fracture/ or exp comminuted fracture/ or exp compression fracture/ or exp device fracture/ or exp distal femur fracture/ or exp distal fibula fracture/ or exp distal radius fracture/ or exp distal tibia fracture/ or exp elbow fracture/ or exp face fracture/ or exp femoral head fracture/ or exp femoral neck fracture/ or exp femur fracture/ or exp femur intertrochanteric fracture/ or exp femur pertrochanteric fracture/ or exp femur shaft fracture/ or exp femur subtrochanteric fracture/ or exp femur trochanteric fracture/ or exp fibula fracture/ or exp finger fracture/ or exp foot fracture/ or exp forearm fracture/ or exp fracture/ or exp fracture dislocation/ or exp fracture external fixation/ or exp fracture fixation/ or exp fracture healing/ or exp fracture healing rate/ or exp fracture immobilization/ or exp fracture infection/ or exp fracture nonunion/ or exp fracture reduction/ or exp fracture risk assessment/ or exp fracture treatment/ or exp fragility fracture/ or exp greenstick fracture/ or exp hand fracture/ or exp hangman's fracture/ or exp hip fracture/ or exp humeral supracondylar fracture/ or exp humerus fracture/ or exp humerus shaft fracture/ or exp insufficiency fracture/ or exp intraarticular fracture/ or exp jaw fracture/ or exp knee fracture/ or exp lateral malleolar fracture/ or exp Le Fort fracture/ or exp lead fracture/ or exp leg fracture/ or exp limb fracture/ or exp Lisfranc fracture/ or exp lumbar spine fracture/ or exp malleolus fracture/ or exp mandible fracture/ or exp maxilla fracture/ or exp medial malleolar fracture/ or exp metacarpal bone fracture/ or exp midface fracture/ or exp multiple fracture/ or exp nose fracture/ or exp odontoid process fracture/ or exp olecranon fracture/ or exp open fracture/ or exp open fracture reduction/ or exp orbit fracture/ or exp orbital floor fracture/ or exp pathologic fracture/ or exp pelvis fracture/ or exp periprosthetic fracture/ or exp phalanx fracture/ or exp pilon fracture/ or exp proximal femur fracture/ or exp proximal humerus fracture/ or exp proximal radius fracture/ or exp radius fracture/ or exp rib fracture/ or exp sacrum fracture/ or exp scaphoid fracture/ or exp shoulder fracture/ or exp skull fracture/ or exp spine fracture/ or exp spiral fracture/ or exp stent fracture/ or exp sternum fracture/ or exp stress fracture/ or exp thoracic fracture/ or exp thoracic spine fracture/ or exp thoracolumbar burst fracture/ or exp thoracolumbar fracture/ or exp tibia fracture/ or exp tibia shaft fracture/ or exp tibial plateau fracture/ or exp tooth fracture/ or exp ulna fracture/ or exp vertebral body fracture/ or exp wrist fracture/ or exp zygoma arch fracture/ or exp zygomaticomaxillary complex fracture/ or exp distal humeral fracture/ or exp freeze fracture/ or exp graft fracture/ or exp hamate fracture/ or exp proximal tibia fracture/ or exp pubis fracture/ or exp skull base fracture/ or exp closed fracture reduction/ or exp joint fracture/ or exp monteggia fracture/ or exp patella fracture/ or exp peri-implant fracture/ or exp atlas fracture/ or exp Barton fracture/ or exp buckle fracture/ or exp cartilage fracture/ or exp Chance fracture/ or exp clay shoveler's fracture/ or exp crush fracture/ or exp depressed skull fracture/ or exp electrode fracture/ or exp endoprosthesis fracture/ or exp epiphysis plate fracture/ or exp fibula shaft fracture/ or exp "fracture of greater trochanter"/ or exp "fracture of lesser trochanter"/ or exp Galeazzi fracture/ or exp guidewire fracture/ or exp Hoffa fracture/ or exp humeral head fracture/ or exp humeral neck fracture/ or exp ilium fracture/ or exp impaction fracture/ or exp Jefferson fracture/ or exp lunate fracture/ or exp orbital roof fracture/ or exp panfacial fracture/ or exp pin fracture/ or exp proximal fibula fracture/ or exp radius shaft fracture/ or exp scapula fracture/ or exp talus fracture/ or exp temporal bone fracture/ or exp thumb fracture/ or exp toe fracture/ or exp trapezium fracture/ or exp triquetrum fracture/ or exp vertebral pedicle fracture/ or exp wire fracture/ or exp capitate fracture/ or exp frontobasal fracture/ or exp posttransplantation fracture/ or exp Smith fracture/ or exp pisiform fracture/ = 517327

3: 1 and 2 = 161

MEDLINE

13/5/2025

1: exp Radiostereometric Analysis/ or radiostereometry.mp. [mp=title, book title, abstract, original title, name of substance word, subject heading word, floating sub-heading word, keyword heading word, organism supplementary concept word, protocol supplementary concept word, rare disease supplementary concept word, unique identifier, synonyms, population supplementary concept word, anatomy supplementary concept word] = 600

2: (Ankle Fracture or Avulsion Fracture or Basilar Skull Fracture or Bicondylar Bicondylar Tibial Plateau Fracture or Bimalleolar Ankle Fracture or Bimalleolar Equivalent Fracture or Bimalleolar Fracture or Bimalleolar Ankle Fracture, Bimalleolar or Blow Out Fracture or Blow-Out Fracture or Blowout Fracture or Bone Fracture or Capitellar Fracture, Humeral or Capitellum Humeri Fracture or Capitellum Humerus Fracture or Cartilage Fracture or Closed Fracture or Closed Fracture Reduction or Closed Fracture Reductions or Closed Reduction, Fracture or Colles Fracture or Colles' Fracture or Comminuted Fracture or Compound Fracture or Compression Fracture or Condyle Fracture, Femoral or Condyles Fracture, Femoral or Crossunited Fracture or Crush Fracture or Depressed Skull Fracture or Dislocation Fracture or Dislocation, Fracture or Dislocation, Galeazzi Fracture or Dislocation, Maisonneuve Fracture or Dislocations, Fracture or Distal Femoral Fracture or Distal Femur Fracture or Distal Forearm Fracture or Distal Humeral Fracture or Distal Humerus Fracture or Distal Radius Fracture or Distal Ulna Fracture or Elbow Fracture or Eminence Fracture, Tibial or Equivalent Fracture, Bimalleolar or Fatigue Fracture or Femoral Condyle Fracture or Femoral Condyles Fracture or Femoral Fracture or Femoral Fracture, Distal or Femoral Fracture, Proximal or Femoral Neck Fracture or Femoral Trochlear Fracture or Femur Fracture, Distal or Femur Fracture, Proximal or Femur Neck Fracture or Femur Trochlear Fracture or Fibula Fracture or Fibula Shaft Fracture or Fixation, Fracture or Fixation, Internal Fracture or Fixation, Intramedullary Fracture or Fixations, Fracture or Fixations, Internal Fracture or Fixations, Intramedullary Fracture or Forearm Fracture, Distal or Fracture Closed Reduction or Fracture Closed Reductions or Fracture Dislocation or Fracture Dislocation, Galeazzi or Fracture Dislocation, Maisonneuve or Fracture Dislocations or Fracture Fixation or Fracture Fixation, Internal or Fracture Fixation, Intramedullary or Fracture Fixations or Fracture Fixations, Internal or Fracture Fixations, Intramedullary or Fracture Healing or Fracture Healings or Fracture Malunion or Fracture Malunions or Fracture Open Reduction or Fracture Open Reductions or Fracture Osteosyntheses or Fracture Osteosynthesis or Fracture Reduction or Fracture Reduction, Closed or Fracture Reduction, Open or Fracture Reductions or Fracture Reductions, Closed or Fracture Strength or Fracture Strengths or "Fracture of Head of Radius" or "Fracture of Neck of Radius" or Fracture, Abnormal Union or Fracture, Ankle or Fracture, Avulsion or Fracture, Basilar Skull or Fracture, Bimalleolar or Fracture, Bimalleolar Ankle or Fracture, Bimalleolar Equivalent or Fracture, Blow Out or Fracture, Blow-Out or Fracture, Blowout or Fracture, Bone or Fracture, Capitellum Humeri or Fracture, Capitellum Humerus or Fracture, Cartilage or Fracture, Closed or Fracture, Colles' or Fracture, Comminuted or Fracture, Compound or Fracture, Compression or Fracture, Crossunited or Fracture, Crush or Fracture, Depressed Skull or Fracture, Dislocation or Fracture, Distal Femoral or Fracture, Distal Femur or Fracture, Distal Forearm or Fracture, Distal Humeral or Fracture, Distal Humerus or Fracture, Distal Radius or Fracture, Distal Ulna or Fracture, Elbow or Fracture, Fatigue or Fracture, Femoral or Fracture, Femoral Condyle or Fracture, Femoral Condyles or Fracture, Femoral Trochlear or Fracture, Femur Trochlear or Fracture, Fibula or Fracture, Fibula Shaft or Fracture, Frontobasilar Skull or Fracture, Galeazzi or Fracture, Greater Tuberosity or Fracture, Growth Plate or Fracture, Hahn-Steinthal or Fracture, Hangman or Fracture, Hangman's or Fracture, Hoffa or Fracture, Humeral or Fracture, Humeral Capitellar or Fracture, Humeral Head or Fracture, Humeral Trochlear or Fracture, Humeri or Fracture, Humerus or Fracture, Humerus Trochlear or Fracture, Insufficiency or Fracture, Intercondylar Humeral or Fracture, Intercondylar Humerus or Fracture, Intra-Articular or Fracture, Intraarticular or Fracture, Jaw or Fracture, Knee or Fracture, Knee Joint or Fracture, Kneecap or Fracture, Kocher-Lorenz or Fracture, Lateral Malleolus or Fracture,

Maisonneuve or Fracture, Malunited or Fracture, Mandibular or Fracture, March or Fracture, Maxillary or Fracture, Medial Malleolus or Fracture, Micro or Fracture, Monteggia's or Fracture, Multiple or Fracture, Non-Depressed Skull or Fracture, Occult or Fracture, Olecranon or Fracture, Olecranon Process or Fracture, Open or Fracture, Orbital or Fracture, Osteoporotic or Fracture, Patella or Fracture, Pathologic or Fracture, Pathological or Fracture, Peri-Implant or Fracture, Periprosthetic or Fracture, Posterior Malleolus or Fracture, Proximal Femoral or Fracture, Proximal Femur or Fracture, Proximal Humeral or Fracture, Proximal Radial or Fracture, Proximal Radius or Fracture, Proximal Ulna or Fracture, Radial or Fracture, Radial Head or Fracture, Radial Neck or Fracture, Radial Styloid or Fracture, Radius or Fracture, Rib or Fracture, Segond or Fracture, Shoulder or Fracture, Skull or Fracture, Smith or Fracture, Spinal or Fracture, Spiral or Fracture, Spontaneous or Fracture, Sprain or Fracture, Stress or Fracture, Tibial or Fracture, Tibial Eminence or Fracture, Tibial Plateau or Fracture, Tibial Spine or Fracture, Tibial Tuberosity or Fracture, Tillaux or Fracture, Toddler's or Fracture, Tooth or Fracture, Torsion or Fracture, Trimalleolar or Fracture, Trimalleolar Ankle or Fracture, Ulna or Fracture, Ununited or Fracture, Wrist or Fracture, Zygomatic or Galeazzi Fracture or Galeazzi Fracture Dislocation or Greater Tuberosity Fracture or Growth Plate Fracture or Hahn Steinthal Fracture or Hahn-Steinthal Fracture or Hangman Fracture or Hangman's Fracture or Hangmans Fracture or Head Fracture, Humeral or Head Fracture, Radial or Healing, Fracture or Healings, Fracture or Hoffa Fracture or Humeral Capitellar Fracture or Humeral Fracture or Humeral Fracture, Distal or Humeral Fracture, Intercondylar or Humeral Fracture, Proximal or Humeral Head Fracture or Humeral Head Fracture Dislocation or Humeral Trochlear Fracture or Humeri Fracture or Humeri Fracture, Capitellum or Humerus Fracture or Humerus Fracture, Capitellum or Humerus Fracture, Distal or Humerus Fracture, Intercondylar or Humerus Trochlear Fracture or Insufficiency Fracture or Intercondylar Humeral Fracture or Intercondylar Humerus Fracture or Internal Fracture Fixation or Internal Fracture Fixations or Intra-Articular Fracture or Intraarticular Fracture or Intramedullary Fracture Fixation or Intramedullary Fracture Fixations or Jaw Fracture or Joint Fracture, Knee or Knee Fracture or Knee Joint Fracture or Kneecap Fracture or Kocher Lorenz Fracture or Kocher-Lorenz Fracture or "Lateral Condylar Fracture of Humerus" or "Lateral Condyle Fracture of Humerus" or "Lateral Epicondylar Fracture of Humerus" or "Lateral Epicondyle Fracture of Humerus" or Lateral Malleolus Fracture or Lateral Tibial Plateau Fracture or LateralMedialMedial Tibial Plateau Fracture or Linear Skull Fracture or Maisonneuve Fracture or Maisonneuve Fracture Dislocation or Malleolus Fracture, Lateral or Malleolus Fracture, Medial or Malleolus Fracture, Posterior or Malunion, Fracture or Malunions, Fracture or Malunited Fracture or Mandibular Fracture or March Fracture or Maxillary Fracture or Medial Malleolus Fracture or Micro Fracture or Monteggia Fracture or Monteggia Fracture Dislocations or Monteggia's Fracture or Monteggias Fracture or Multiple Fracture or Neck Fracture, Radial or Non Depressed Skull Fracture or Non-Depressed Skull Fracture or Non-DepressedSkull Fracture, Non Depressed or OSTEOSYN FRACTURE or OSTEOSYN FRACTURE INTRAMEDULLARY or Occult Fracture or Olecranon Fracture or Olecranon Process Fracture or Open Fracture or Open Fracture Reduction or Open Fracture Reductions or Open Reduction, Fracture or Open Reductions, Fracture or Orbital Fracture or Osteoporotic Fracture or Osteosyntheses, Fracture or Osteosynthesis, Fracture or Osteosynthesis, Fracture, Intramedullary or Out Fracture, Blow or Patella Fracture or Pathologic Fracture or Pathological Fracture or Peri-Implant Fracture or Periprosthetic Fracture or Posterior Malleolus Fracture or Proximal Femoral Fracture or Proximal Femur Fracture or Proximal Humeral Fracture or Proximal Radial Fracture or Proximal Radius Fracture or Proximal Ulna Fracture or Radial Fracture or Radial Fracture, Proximal or Radial Head Fracture or Radial Neck Fracture or Radial Styloid Fracture or Radius Fracture or Radius Fracture, Distal or Radius Fracture, Proximal or Reduction, Fracture or Reduction, Fracture Closed or Reduction, Fracture Open or Reductions, Fracture or Reductions, Fracture Closed or Reductions, Fracture Open or Rib Fracture or Segond Fracture or Shaft Fracture, Fibula or Shoulder Fracture or Skull Fracture or Skull Fracture, Basilar or Skull Fracture, Basilar, Childhood or Skull Fracture, Compound Depressed or Skull Fracture, Depressed or Skull Fracture, Frontobasilar or Skull Fracture, Linear or Skull Fracture, Non-Depressed or Skull Fracture, Transphenoid Basilar or Smith Fracture or Spinal Fracture or Spine Fracture, Tibial or Spiral Fracture or Spontaneous Fracture or Sprain Fracture or Strength, Fracture or Strengths, Fracture or Stress Fracture or Styloid Fracture, Radial or Supracondylar Distal Humeral Fracture or Supracondylar Supracondylar Distal Humerus Fracture or Tibial Eminence Fracture or Tibial Fracture or Tibial Plateau Fracture or Tibial Spine Fracture or Tibial Tuberosity Fracture or Tillaux Fracture or Toddler Fracture or Toddler's Fracture or Toddlers Fracture or Tooth Fracture or Torsion

Fracture or Trimalleolar Ankle Fracture or Trimalleolar Fracture or TrimalleolarAnkle Fracture, Trimalleolar or Trochlear Fracture, Femoral or Trochlear Fracture, Femur or Trochlear Fracture, Humeral or Trochlear Fracture, Humerus or Tuberosity Fracture, Tibial or Ulna Fracture or Ulna Fracture, Distal or Ulna Fracture, Proximal or Ununited Fracture or Wrist Fracture or Zygomatic Fracture).mp. = 146927

3: 1 and 2 = 25
